# The Ear—Its Anatomy, Physiology and Diseases

**Published:** 1878-01

**Authors:** 


The Ear; Its Anatomy, Physiology and Diseases. A Practical Treatise for the use of Medical Students and Practitioners. By Charles H. Burnett, A.M., M.D., Aural Surgeon to the Presbyterian Hospital, etc. Philadelphia; Henry C. Lea. 1877.

This is a new work of 600 pages, and is, doubtless, an exhaustive treatise on otology. Having been received too late for perusal in order to be able to write more at length of the book, we can only say, from a glance over its pages, that we have not been more pleased with the appearance and arrangement of subjects than in this. We are pleased, also, to see this evidence of sufficient ability and energy at home to supply the demand for standard works on special or general subjects, without republication from European authors, because of their popularity twenty years ago.

